# Supplementary material for: Magnitude and predictors of HIV-Drug resistance in Africa: A protocol for systematic review and meta-analysis
Source: PLoS One. 2022 Apr 20;17(4):e0267159. doi: 10.1371/journal.pone.0267159 (PMC9020684; doi:10.1371/journal.pone.0267159)
Supplement: S2 File — (DOC) [file pone.0267159.s002.doc]

**S2 File: Search strategy for major databases**

1. PubMed

| S/No | **Search term/logic** | **Result** |
| --- | --- | --- |
| 1 | ("HIV Infections"[mh] or "HIV"[mh] or "hiv-1" or "HIV-2" or "HIV Infect*" or "Human Immunodeficiency Virus"[tiab] or "Human Immuno-deficiency Virus"[tiab] or "Human Immune-deficiency Virus"[tiab]) |  |
| 2 | ("HIV Infections" OR "HIV" OR "HIV-1" OR "HIV-2" OR"HIV Infect*" OR "Human Immunodeficiency Virus" OR "Human Immuno-deficiency Virus" OR "Human Immune-deficiency Virus") |  |
| 3 | #1 OR #2 |  |
| 4 | ("Drug resist*" or "Transmitted resistance" or "TDR" or "ADR" or "PDR" or HIVDR) |  |
| 5 | (mutant* OR gene* OR genom* OR suppress* OR protein* OR mutat* OR alter* OR damage* OR aberration* OR genotyp* OR Nucleo?ide reverse-transcriptase inhibitor* OR non?Nucleo?ide reverse-transcriptase inhibitor* OR NNRTI* OR NRTI* OR Protease inhibitor* OR boosted protease inhibitor* OR bPI or Allelic Imbalance OR Base Pair Mismatch OR Condon OR Mutagen* OR Sequence Deletion OR Sequence inversion OR quasispecies) |  |
| 6 | (Africa* or sub-Saharan Africa* or Algeria* or Angola* or Benin or Botswana* or "Burkina Faso" or Burundi* or "Cabo Verd*" or "Cape Verd*" or Cameroon* or "Central African*" or Chad* or Congo or "Cote d'Ivoire" or "Ivory Coast" or Djibouti or Egypt or Eritrea* or Ethiopia* or Gabon* or Gambia* or Ghana* or Guinea* or Kenya* or Liberia* or Libya* or Madagascar* or Malawi* or Mali or Morocc* or Mozambique or Namibia* or Niger* or Nigeria* or Rwanda* or Senegal* or " Sierra Leone* " or Somalia* or " South Africa* " or Sudan* or Swaziland* or Tanzania* or Togo* or Tonga* or Tunisia* or Uganda* or Zambia* or Zimbabw* or Lesotho or Maurit*) |  |
| 7 | #3 AND #4 AND #5 AND #6 |  |
| 8 | Filters applied: Clinical Conference, Clinical Study, Clinical Trial, Comparative Study, Controlled Clinical Trial, Corrected and Republished Article, Journal Article, Multicenter Study, Observational Study, Randomized Controlled Trial, Humans, English, from 2000/1/1 - 2021/30/26. |  |

1. **Medline Ovid**

| **S/N** | **Search terms/logics** | **Result** |
| --- | --- | --- |
| 1 | exp HIV Infections/ |  |
| 2 | (HIV or "HIV-1" or "HIV-2" or "Human Immunodeficiency Virus" or "Human Immuno-deficiency Virus" or "Human Immune-deficiency Virus" or AIDS or "acquired immunodeficiency syndrome").tw,kf. |  |
| 3 | or/1-2 |  |
| 4 | drug resistance/ or exp drug resistance, viral/ or drug resistance, multiple, viral/ |  |
| 5 | ("Drug resist*" or "Transmitted resistance" or "TDR" or "ADR" or "PDR" or HIVDR).tw,kw |  |
| 6 | or/4-5 |  |
| 7 | exp Mutation/ |  |
| 8 | (mutant* OR gene* or genom* OR suppress* OR protein* OR mutat* OR alter* OR damage* OR aberration* OR genotyp* OR Nucleo?ide reverse-transcriptase inhibitor* OR non?Nucleo?ide reverse-transcriptase inhibitor* OR NNRTI* OR NRTI* OR Protease inhibitor* OR boosted protease inhibitor* OR bPI OR Allelic Imbalance OR Base Pair Mismatch OR Condon OR Mutagen* OR Sequence Deletion OR Sequence inversion OR quasispecies).tw,kw |  |
| 9 | or/7-8 |  |
| 10 | exp Africa/ |  |
| 11 | (africa* or sub-Saharan Africa* or Algeria* or Angola* or Benin or Botswana* or "Burkina Faso" or Burundi* or "Cabo Verd*" or "Cape Verd*" or Cameroon* or "Central African*" or Chad* or Congo or "Cote d'Ivoire" or "Ivory Coast" or Djibouti or Egypt or Eritrea* or Ethiopia* or Gabon* or Gambia* or Ghana* or Guinea* or Kenya* or Liberia* or Libya* or Madagascar* or Malawi* or Mali or Morocc* or Mozambique or Namibia* or Niger* or Nigeria* or Rwanda* or Senegal* or " Sierra Leone* " or Somalia* or " South Africa* " or Sudan* or Swaziland* or Tanzania* or Togo* or Tonga* or Tunisia* or Uganda* or Zambia* or Zimbabw* or Lesotho or Maurit*).tw,kw |  |
| 12 | or/10-11 |  |
| 13 | 3 and 6 and 9 and 12 |  |
| 14 | limit 13 to (english language and humans and yr="2000 -Current" and (clinical conference or clinical study or clinical trial or comparative study or controlled clinical trial or "corrected and republished article" or journal article or multicenter study or observational study or preprint or randomized controlled trial)) |  |

1. CINAHL

| **S/N** | **Query** | **Result** |
| --- | --- | --- |
| S1 | (MH "HIV infections+") |  |
| S2 | TI ("HIV infect*" OR HIV or "HIV-1" or "HIV-2" or "Human Immunodeficiency Virus" or "Human Immuno-deficiency Virus" or "Human Immune-deficiency Virus" or AIDS or "acquired immunodeficiency syndrome") OR AB ( "HIV infect*" OR HIV or "HIV-1" or "HIV-2" or "Human Immunodeficiency Virus" or "Human Immuno-deficiency Virus" or "Human Immune-deficiency Virus" or AIDS or "acquired immunodeficiency syndrome") |  |
| S3 | S1 OR S2 |  |
| 54 | (MH "Drug Resistance") |  |
| S5 | (MH "Drug Resistance, Multiple") |  |
| S6 | TI ( "Drug resist*" OR "Transmitted resistance" OR "Transmitted Drug resist*" OR "TDR" OR "Acquired Drug Resist*" OR "ADR" OR "Pretreatment Drug Resist*" OR "Pre-treatment Drug Resist*" OR "PDR" OR "HIVDR" ) OR AB ( "Drug resist*" OR "Transmitted resistance" OR "Transmitted Drug resist*" OR "TDR" OR "Acquired Drug Resist*" OR "ADR" OR "Pretreatment Drug Resist*" OR "Pre-treatment Drug Resist*" OR "PDR" OR "HIVDR" ) |  |
| S7 | S4 OR S5 OR S6 |  |
| S8 | (MH "Mutation+") |  |
| S9 | TI (Mutant* OR gene* OR genom* OR suppress* OR protein* OR mutat* OR alter* OR damage* OR aberration* OR genotyp* OR "Nucleo?ide reverse-transcriptase inhibitor*" OR "non?Nucleo?ide reverse-transcriptase inhibitor*" OR NNRTI* OR NRTI* OR "Protease inhibitor*" OR "PI" OR "boosted protease inhibitor*" OR "bPI" or "Allelic Imbalance" OR "Base Pair Mismatch" OR condon OR Mutagen* OR "Sequence Deletion” OR "Sequence inversion” OR "quasispecies" ) OR AB (Mutant* OR gene* OR genom* OR suppress* OR protein* OR mutat* OR alter* OR damage* OR aberration* OR genotyp* OR "Nucleo?ide reverse-transcriptase inhibitor*" OR "non?Nucleo?ide reverse-transcriptase inhibitor*" OR NNRTI* OR NRTI* OR "Protease inhibitor*"OR "PI" OR "boosted protease inhibitor*" OR "bPI" or "Allelic Imbalance" OR "Base Pair Mismatch "OR condon OR Mutagen* OR "Sequence Deletion” OR "Sequence inversion” OR "quasispecies") |  |
| S10 | S8 OR S9 |  |
| S11 | (MH "Africa+") |  |
| S12 | TI ( Africa* OR "Sub-Saharan Africa*" OR "Northern Africa*" OR "Africa south of the Sahara" OR "Central Africa*" OR "Eastern Africa*" OR "Southern Africa*" OR "Western Africa*" OR Algeria* OR Angola* OR Benin OR Botswana* OR "Burkina Faso" OR Burundi* OR "Cabo Verd*" OR "Cape Verd*" OR Cameroon* OR "Central African*" OR Chad* OR Congo OR "Cote d'Ivoire" OR "Ivory Coast" OR Djibouti or Egypt OR Eritrea* OR Ethiopia* OR Gabon* OR Gambia* OR Ghana* OR Guinea* OR Kenya* OR Liberia* OR Libya* OR Madagascar* OR Malawi* OR Mali OR Morocc* OR Mozambique OR Namibia* OR Niger* OR Nigeria* OR Rwanda* OR Senegal* OR " Sierra Leone* " OR Somalia* OR " South Africa* " OR Sudan* OR Swaziland* OR Tanzania* OR Togo* OR Tonga* OR Tunisia* OR Uganda* OR Zambia* OR Zimbabw* OR Lesotho OR Maurit* ) OR AB ( Africa* OR "Sub-Saharan Africa*" OR "Northern Africa*" OR "Africa south of the sahara" OR "Central Africa*" OR "Eastern Africa*" OR "Southern Africa*" OR "Western Africa*" OR Algeria* OR Angola* OR Benin OR Botswana* OR "Burkina Faso" OR Burundi* OR "Cabo Verd*" OR "Cape Verd*" OR Cameroon* OR "Central African*" OR Chad* OR Congo OR "Cote d'Ivoire" OR "Ivory Coast" OR Djibouti or Egypt OR Eritrea* OR Ethiopia* OR Gabon* OR Gambia* OR Ghana* OR Guinea* OR Kenya* OR Liberia* OR Libya* OR Madagascar* OR Malawi* OR Mali OR Morocc* OR Mozambique OR Namibia* OR Niger* OR Nigeria* OR Rwanda* OR Senegal* OR " Sierra Leone* " OR Somalia* OR " South Africa* " OR Sudan* OR Swaziland* OR Tanzania* OR Togo* OR Tonga* OR Tunisia* OR Uganda* OR Zambia* OR Zimbabw* OR Lesotho OR Maurit*) |  |
| S13 | S11 OR S12 |  |
| S14 | S3 AND S7 AND S10 AND S13 |  |
| S15 | S3 AND S7 AND S10 AND S13  Limited to 2000-2021 and Academic journals and dissertation |  |

1. **Scopus**

| **Balloon operator** | **Search term/logic** | **Result** |
| --- | --- | --- |
|  | ("HIV infect*" OR "HIV" OR "HIV-1" OR "HIV-2" OR "Human Immunodeficiency Virus" OR "Human Immuno-deficiency Virus" OR "Human Immune-deficiency Virus" OR AIDS OR "acquired immunodeficiency syndrome") |  |
| **AND** | ("Drug resist*" OR "Transmitted resistance" OR "Transmitted Drug resist*" OR TDR OR "Acquired Drug Resist*" OR "ADR" OR "Pretreatment Drug Resist*" OR "Pre-treatment Drug Resist*" OR "PDR" OR "HIVDR") |  |
| **AND** | (mutant* OR gene* or genom* OR suppress* OR protein* OR mutat* OR alter* OR damage* OR aberration* OR genotyp* OR "Nucleo?ide reverse-transcriptase inhibitor*" OR "non?Nucleo?ide reverse-transcriptase inhibitor*" OR NNRTI* OR NRTI* OR "Protease inhibitor*"OR "PI" OR "boosted protease inhibitor*" OR "bPI" or "Allelic Imbalance" OR "Base Pair Mismatch "OR condon OR Mutagen* OR "Sequence Deletion” OR "Sequence inversion” OR "quasispecies") |  |
| **AND** | (Africa* OR "Sub-Saharan Africa*" OR "Northern Africa*" OR "Africa south of the sahara" OR "Central Africa*" OR "Eastern Africa*" OR "Southern Africa*" OR "Western Africa*" OR Algeria* OR Angola* OR Benin OR Botswana* OR "Burkina Faso" OR Burundi* OR "Cabo Verd*" OR "Cape Verd*" OR Cameroon* OR "Central African*" OR Chad* OR Congo OR "Cote d'Ivoire" OR "Ivory Coast" OR Djibouti or Egypt OR Eritrea* OR Ethiopia* OR Gabon* OR Gambia* OR Ghana* OR Guinea* OR Kenya* OR Liberia* OR Libya* OR Madagascar* OR Malawi* OR Mali OR Morocc* OR Mozambique OR Namibia* OR Niger* OR Nigeria* OR Rwanda* OR Senegal* OR " Sierra Leone* " OR Somalia* OR " South Africa* " OR Sudan* OR Swaziland* OR Tanzania* OR Togo* OR Tonga* OR Tunisia* OR Uganda* OR Zambia* OR Zimbabw* OR Lesotho OR Maurit*) |  |
|  | **Results:** |  |
|  | AND  ( LIMIT-TO ( PUBYEAR ,  2021 )  OR  LIMIT-TO ( PUBYEAR ,  2020 )  OR  LIMIT-TO ( PUBYEAR ,  2019 )  OR  LIMIT-TO ( PUBYEAR ,  2018 )  OR  LIMIT-TO ( PUBYEAR ,  2017 )  OR  LIMIT-TO ( PUBYEAR ,  2016 )  OR  LIMIT-TO ( PUBYEAR ,  2015 )  OR  LIMIT-TO ( PUBYEAR ,  2014 )  OR  LIMIT-TO ( PUBYEAR ,  2013 )  OR  LIMIT-TO ( PUBYEAR ,  2012 )  OR  LIMIT-TO ( PUBYEAR ,  2011 )  OR  LIMIT-TO ( PUBYEAR ,  2010 )  OR  LIMIT-TO ( PUBYEAR ,  2009 )  OR  LIMIT-TO ( PUBYEAR ,  2008 )  OR  LIMIT-TO ( PUBYEAR ,  2007 )  OR  LIMIT-TO ( PUBYEAR ,  2006 )  OR  LIMIT-TO ( PUBYEAR ,  2005 )  OR  LIMIT-TO ( PUBYEAR ,  2004 )  OR  LIMIT-TO ( PUBYEAR ,  2003 )  OR  LIMIT-TO ( PUBYEAR ,  2002 )  OR  LIMIT-TO ( PUBYEAR ,  2001 )  OR  LIMIT-TO ( PUBYEAR ,  2000 ) )  AND  ( LIMIT-TO ( DOCTYPE ,  "ar" )  OR  LIMIT-TO ( DOCTYPE ,  "cp" )  OR  LIMIT-TO ( DOCTYPE ,  "sh" ) )  AND  ( LIMIT-TO ( LANGUAGE ,  "English" ) )  AND  ( LIMIT-TO ( SRCTYPE ,  "j" )  OR  LIMIT-TO ( SRCTYPE ,  "p" )  OR  LIMIT-TO ( SRCTYPE ,  "Undefined" ) ) |  |

1. Web of Science

| **Balloon operator** | **Query** | **Result** |
| --- | --- | --- |
|  | ("HIV infect*" OR "HIV" OR "HIV-1" OR "HIV-2" OR "Human Immunodeficiency Virus" OR "Human Immuno-deficiency Virus" OR "Human Immune-deficiency Virus" OR AIDS OR "acquired immunodeficiency syndrome") |  |
| **AND** | ("Drug resist*" OR "Transmitted resistance" OR "Transmitted Drug resist*" OR "TDR" OR "Acquired Drug Resist*" OR "ADR" OR "Pretreatment Drug Resist*" OR "Pre-treatment Drug Resist*" OR "PDR" OR "HIVDR") |  |
| **AND** | (Mutant* OR gene* OR genom* OR suppress* OR protein* OR mutat* OR alter* OR damage* OR aberration* OR genotyp* OR "Nucleo?ide reverse-transcriptase inhibitor*" OR "non?Nucleo?ide reverse-transcriptase inhibitor*" OR NNRTI* OR NRTI* OR "Protease inhibitor*"OR "PI" OR "boosted protease inhibitor*" OR "bPI" or "Allelic Imbalance" OR "Base Pair Mismatch "OR condon OR Mutagen* OR "Sequence Deletion” OR "Sequence inversion” OR "quasispecies") |  |
| **AND** | (Africa* OR "Sub-Saharan Africa*" OR "Northern Africa*" OR "Africa south of the sahara" OR "Central Africa*" OR "Eastern Africa*" OR "Southern Africa*" OR "Western Africa*" OR Algeria* OR Angola* OR Benin OR Botswana* OR "Burkina Faso" OR Burundi* OR "Cabo Verd*" OR "Cape Verd*" OR Cameroon* OR "Central African*" OR Chad* OR Congo OR "Cote d'Ivoire" OR "Ivory Coast" OR Djibouti or Egypt OR Eritrea* OR Ethiopia* OR Gabon* OR Gambia* OR Ghana* OR Guinea* OR Kenya* OR Liberia* OR Libya* OR Madagascar* OR Malawi* OR Mali OR Morocc* OR Mozambique OR Namibia* OR Niger* OR Nigeria* OR Rwanda* OR Senegal* OR " Sierra Leone* " OR Somalia* OR " South Africa* " OR Sudan* OR Swaziland* OR Tanzania* OR Togo* OR Tonga* OR Tunisia* OR Uganda* OR Zambia* OR Zimbabw* OR Lesotho OR Maurit*) |  |
|  | (((TS=(("HIV infect*" OR "HIV" OR "HIV-1" OR "HIV-2" OR "Human Immunodeficiency Virus" OR "Human Immuno-deficiency Virus" OR "Human Immune-deficiency Virus" OR AIDS OR "acquired immunodeficiency syndrome"))) AND TS=(("Drug resist*" OR "Transmitted resistance" OR "Transmitted Drug resist*" OR "TDR" OR "Acquired Drug Resist*" OR "ADR" OR "Pretreatment Drug Resist*" OR "Pre-treatment Drug Resist*" OR "PDR" OR "HIVDR"))) AND TS=((Mutant* OR gene* OR genom* OR suppress* OR protein* OR mutat* OR alter* OR damage* OR aberration* OR genotyp* OR "Nucleo?ide reverse-transcriptase inhibitor*" OR "non?Nucleo?ide reverse-transcriptase inhibitor*" OR NNRTI* OR NRTI* OR "Protease inhibitor*"OR "PI" OR "boosted protease inhibitor*" OR "bPI" or "Allelic Imbalance" OR "Base Pair Mismatch "OR condon OR Mutagen* OR "Sequence Deletion” OR "Sequence inversion” OR "quasispecies"))) AND TS=((Africa* OR "Sub-Saharan Africa*" OR "Northern Africa*" OR "Africa south of the sahara" OR "Central Africa*" OR "Eastern Africa*" OR "Southern Africa*" OR "Western Africa*" OR Algeria* OR Angola* OR Benin OR Botswana* OR "Burkina Faso" OR Burundi* OR "Cabo Verd*" OR "Cape Verd*" OR Cameroon* OR "Central African*" OR Chad* OR Congo OR "Cote d'Ivoire" OR "Ivory Coast" OR Djibouti or Egypt OR Eritrea* OR Ethiopia* OR Gabon* OR Gambia* OR Ghana* OR Guinea* OR Kenya* OR Liberia* OR Libya* OR Madagascar* OR Malawi* OR Mali OR Morocc* OR Mozambique OR Namibia* OR Niger* OR Nigeria* OR Rwanda* OR Senegal* OR " Sierra Leone* " OR Somalia* OR " South Africa* " OR Sudan* OR Swaziland* OR Tanzania* OR Togo* OR Tonga* OR Tunisia* OR Uganda* OR Zambia* OR Zimbabw* OR Lesotho OR Maurit*)) |  |
|  | **Results:** |  |
|  | Filtered: year, article type, langue and other relevant |  |
