# Supplementary material for: Magnitude and predictors of HIV-Drug resistance in Africa: A protocol for systematic review and meta-analysis
Source: PLoS One. 2022 Apr 20;17(4):e0267159. doi: 10.1371/journal.pone.0267159 (PMC9020684; doi:10.1371/journal.pone.0267159)
Supplement: Attachment — Submitted filename: renamed_35251.doc [file pone.0267159.s003.doc]

**Authors Response to Reviewers’ Comments (PONE-D-21-32189)**

**PONE-D-21-32189**

Magnitude and predictors of HIV-Drug Resistance in Africa: a protocol for Systematic Review and Meta-analysis

PLOS ONE

Date: 19/03/2022

**Response to Editor**

Additional Editor Comments:

Please address the several minor issues raised by the reviewers

[Note: HTML markup is below. Please do not edit.]

Reviewers' comments:

**Reviewer's Responses to Questions**

**Response**: Thank you for the timely editorial assessment. We addressed the review comments raised by reviewers.

**Comments to the Author**

1. Does the manuscript provide a valid rationale for the proposed study, with clearly identified and justified research questions?

The research question outlined is expected to address a valid academic problem or topic and contribute to the base of knowledge in the field.

Reviewer #1: Yes

Reviewer #2: Yes

**Response**: Thank you

 2. Is the protocol technically sound and planned in a manner that will lead to a meaningful outcome and allow testing the stated hypotheses?

The manuscript should describe the methods in sufficient detail to prevent undisclosed flexibility in the experimental procedure or analysis pipeline, including sufficient outcome-neutral conditions (e.g. necessary controls, absence of floor or ceiling effects) to test the proposed hypotheses and a statistical power analysis where applicable. As there may be aspects of the methodology and analysis which can only be refined once the work is undertaken, authors should outline potential assumptions and explicitly describe what aspects of the proposed analyses, if any, are exploratory.

Reviewer #1: Yes

Reviewer #2: Yes

**Response**: Thank you

3. Is the methodology feasible and described in sufficient detail to allow the work to be replicable?


Descriptions of methods and materials in the protocol should be reported in sufficient detail for another researcher to reproduce all experiments and analyses. The protocol should describe the appropriate controls, sample size calculations, and replication needed to ensure that the data are robust and reproducible.

Reviewer #1: Yes

Reviewer #2: Yes

**Response**: Thank you

4. Have the authors described where all data underlying the findings will be made available when the study is complete?

The [PLOS Data policy](https://journals.plos.org/plosone/s/materials-and-software-sharing) requires authors to make all data underlying the findings described in their manuscript fully available without restriction, with rare exception, at the time of publication. The data should be provided as part of the manuscript or its supporting information, or deposited to a public repository. For example, in addition to summary statistics, the data points behind means, medians and variance measures should be available. If there are restrictions on publicly sharing data—e.g. participant privacy or use of data from a third party—those must be specified.

Reviewer #1: Yes

Reviewer #2: Yes

**Response**: Thank you

5. Is the manuscript presented in an intelligible fashion and written in standard English?

PLOS ONE does not copyedit accepted manuscripts, so the language in submitted articles must be clear, correct, and unambiguous. Any typographical or grammatical errors should be corrected at revision, so please note any specific errors here.

Reviewer #1: No

**Response:** The protocol is edited for clarity and language

Reviewer #2: Yes

**Response**: Thank you

**Response to reviewer #1**

**Response**: The authors would like to thank the reviewers for the scientific and technical comments which would improve the protocol.

Reviewer #1: Thank you for the efforts towards research and academia. However, I noted the following;
1. Introduction, 4th paragraph, 10th line, 1st word: "sab-saharan" instead of sub-saharan

**Response:** Thank you for the prudence in reviewing the protocol. We corrected as suggested

2. Data extraction, 3rd last line, 5th word: "mutilations" instead of mutations

**Response:** Thank you for the critical review. We corrected as suggested

3. Discussion, 1st paragraph, 6th last line Joint United Nations Program on HIV/AIDS -"USAIDS" instead of UNAIDS

1st paragraph last line ART "regiments" instead of ART regimens

**Response:** Thank you for the valuable checking of typos. We corrected as suggested

4. Limitations, 2nd last line "HIDR" instead of HIVDR

**Response:** Thank you for the valuable checking of typos. We corrected as suggested

**Response to reviewer #2**

**Response**: The authors would like to thank the reviewers for the scientific and technical comments which would improve the protocol.

**Reviewer #2:**

1. Multiple claims in the manuscript need citations. For example
(a) The resistance to first-line ART drugs would also affect effectiveness of second-line ART drug.

Response: Thank you.

We supported this argument with a subsequent description thereafter citing references stepwise (Ref 19 -24). We did so because we believed that once the argument is supported by subsequent statement(s), the use of citation for the umbrella sentence is not a must. But, for this revision of the manuscript, we cited references as you suggested to do so (see Paragraph #3).

(b) In Africa, its prevalence has been raising. According to the 2019 WHO report, in six African countries that participated in national HIVDR surveillance, the prevalence is high in children and adults.

**Response**: Thank you.

Two additional references supporting the argument are added in this version of the protocol. Besides, those citation aftermath of these sentence support the arguments that the authors stated (see paragraph #3 of the introduction section).

(c) The world health organization (WHO) reported that up to 26% of people initiating treatment are infected with a virus carrying resistance to first-line antiretroviral drugs,

**Response**: The reference #10 cited after the immediate sentence is for both statements. “The …….WHO reported that up to 26%..........carrying resistance to first-line antiretroviral drugs. The level of HIVDR is higher ………….. up to 69% (10).” If it is a must cite the reference for both sentence separately, we can and it is so in this version. In addition to reference #10, we supported the argument with reference #11 cited in this revision (see paragraph #3 of introduction). .

(d) At the end of 2020, 37.6 million (95% uncertainty level: 30.2 - 45 million) people were living with HIV, of which more than two-thirds were in the African WHO region.

**Response:** Thank you. The evidence is obtained from ‘WHO HIV/AIDS key facts’ website cited in the document after taking the preceding statement next to this sentence. “……in African WHO Region. Moreover, one and half million people were newly infected, and more than half a million people died from HIV-related causes in 2020 ”. If it is a must to cite evidence for every sentence, we can cite reference #1 for this sentence, and we did so.

2. Some minor typos. Change sab-sahara to sub-sahara, also change 42,7% to 42.7%. Revise the whole manuscript to remove some typos errors.

**Response:**

Thank you. We have made corrections and editions based on the reviewers’ comments.

**Additional information to editor & reviewers:**

In this revised version of the protocol, we included supplementary file which showed the search strategies being used for article retrieval from major databases (see S3 file).

Sincerely,

Mulugeta Melku, Corresponding authors
